# The Inconvenience of Hats

**Published:** 1904-09

**Authors:** 


### The Inconvenience of Hats.

FROM an article bearing the above title, which appears in *Cosmos* (Paris, July 16), we learn that the new fashion of going without a hat is not limited to this country. According to the writer, it has everything, hygienically, in its favor, and the arguments against it need only be stated to be refuted. He says:

The mass of hair that covers the top of the head is a feature of the human race in both sexes, and appears to be one of its most stable physical characteristics. Nevertheless, long observation is unnecessary to prove that this characteristic is weakening, and that the vigor of the hair is decreasing in man. Now a question presents itself: is this due to a transformation of the species or must we attribute the fact to man's habits? This second solution appears to be correct, which is consoling, since it allows us to hope that the evil may be checked. This loss of hair that has become more striking from one generation to another by heredity, is due, according to some scientists, to the habit of covering the head.

This habit must affect the hair injuriously in three ways: (1) by depriving it of the life-giving light of the sun, of free ventilation, and of the movement of the hairs by air-currents; (2) by pressure on the small arteries of the scalp, which bring nourishment to the hair; (3) finally, because all head-coverings are an excellent culture-medium for microbes, and facilitate their development. In fact, the hat, since it prevents the germicidal action of the sun's rays and the movement of the air, and retains on the head the heat and moisture of the enclosed air, offers all the most favorable conditions for obtaining a culture of microorganisms. Furthermore, it is well recognised that the chief causes of baldness are the microbian affections of the scalp, which destroy the sebaceous glands.

We may, then, suppose that it is the custom of covering the head that diminishes, little by little, the vigor of the hair. Although this is not absolutely proved, it is infinitely probable, and in any case it would cost nothing to try a change in the present fashion. This change is absolutely desirable, especially for men, for with women, besides the fact that their hats cover only part of the hair, they are generally lighter; the preservation of the hair for the species is due to the women alone, the men counting for nothing in the matter. . . . . .

The promoters of this reform are meeting, at the outset, with certain objections: (1) to uncover the head may bring on colds, neuralgia and rheumatism. They answer that colds, catarrh, and the like, are of microbian origin and can not come from the scalp; (2) as for neuralgia and rheumatism, they are convinced that if the habit of leaving the head uncovered is adopted in youth, these troubles will not follow. In fact, they say, the uncovered parts of the head are not subject to them any more than the covered part—less perhaps; (3) so far as the incontestable danger of exposing the bare head in the sun is concerned, there are many ways of avoiding this without smothering the scalp; (4) the fear that septic bodies may be deposited on the uncovered parts, especially in cities, certainly deserves consideration; but care in the toilet will enable us to escape the consequences; (5) finally, the fear lest the hair should be injured by sun, wind, or cold has no serious basis, since unprotected parts of the head are covered with vigorous hair."—Translation made for *The Literary Digest.*

